# Protein localization prediction using random walks on graphs

**DOI:** 10.1186/1471-2105-14-S8-S4

**Published:** 2013-05-09

**Authors:** Xiaohua Xu, Lin Lu, Ping He, Ling Chen

**Affiliations:** 1 Department of Computer Science, Yangzhou University, Yangzhou 225009, China

## Abstract

**Background:**

Understanding the localization of proteins in cells is vital to characterizing their functions and possible interactions. As a result, identifying the (sub)cellular compartment within which a protein is located becomes an important problem in protein classification. This classification issue thus involves predicting labels in a dataset with a limited number of labeled data points available. By utilizing a graph representation of protein data, random walk techniques have performed well in sequence classification and functional prediction; however, this method has not yet been applied to protein localization. Accordingly, we propose a novel classifier in the site prediction of proteins based on random walks on a graph.

**Results:**

We propose a graph theory model for predicting protein localization using data generated in yeast and gram-negative (Gneg) bacteria. We tested the performance of our classifier on the two datasets, optimizing the model training parameters by varying the laziness values and the number of steps taken during the random walk. Using 10-fold cross-validation, we achieved an accuracy of above 61% for yeast data and about 93% for gram-negative bacteria.

**Conclusions:**

This study presents a new classifier derived from the random walk technique and applies this classifier to investigate the cellular localization of proteins. The prediction accuracy and additional validation demonstrate an improvement over previous methods, such as support vector machine (SVM)-based classifiers.

## Background

Protein localization is a general a term that refers to the study of where proteins are located within the cell. In many cases, proteins cannot perform their designated function until they are transported to the proper location at the appropriate time. Improper localization of proteins can exert a significant impact on cellular processes or on the entire organism. Therefore, a central issue for biologists is to predict the (sub)cellular localization of proteins[1-3], which has implications for the functions and interactions[4,5] of proteins.

With the development of new approaches in computer science, coupled with an improved dataset of proteins with known localization, computational tools can now provide fast and accurate localization predictions for many organisms as an alternative to laboratory-based methods. Therefore, many studies have begun to address this issue. To predict the cellular localization of proteins, soon after their proposal of a probabilistic classification system to identify 336 E.coli proteins and the 1484 yeast proteins [6], Paul Horton and Kenta Nakai [7] also compared their specifically designed probabilistic model with three other classifiers on the same datasets: the k-nearest-neighbor (kNN) classifier, the binary decision tree classifier, and the naive Bayes classifier. The resulting accuracy using stratified cross-validation showed that the kNN classifier performed better than the other methods, with an accuracy of approximately 60% for 10 yeast classes and 86% for 8 E. coli classes.

Feng [8] presented an overview about the prediction of protein subcellular localization, and in 2004, Donnes and Hoglund [9] introduced past and current work on this type of prediction as well as a guideline for future studies. Chou and Shen [10] summarized the more recent advances in the prediction of protein subcellular localization up to 2007. A variety of artificial intelligence technologies [11-15] have now been developed, including neural networks, the covariant discriminate algorithm, hidden Markov models (HMMs), Decision Tree and support vector machines (SVMs). Among these methods, the SVMs are always considered as a powerful algorithm for supervised learning.

Besides, there are other methods proposed too, like the YLoc tool implemented by Briesemeister et al. [16] and the PROlocalizer [17] which integrated web service to aid the prediction. Recently, the random-walk-on-graph technique [18-20] has been applied to biological questions such as the classification of proteins into functional and structural classes based on their amino acid sequences. Weston et al. presented a random-walk kernel based on PSI-BLAST E-values [21] for protein remote homology detection. Min et al. [22] applied the convex combination algorithm to approximate the random-walk kernel with optimal random steps and applied this approach to classify protein sequence. Freschi et al. [23] proposed a random walk ranking algorithm to predict protein functions from interaction networks. Random walks are closely linked to Markov chains, which inspired Yuan [24] to apply a first-order Markov chain and extend the residue pair probability to higher-order models to predict protein subcellular locations. Garagea et al. [25] also presented a semi-supervised method for prediction using abstraction augmented Markov models.

This study introduces a novel random walk method for protein subcellular localization based on amino acid composition. By mapping the protein data into a weighted and partially labeled graph where each node represents a protein sequence, we implemented a random walk classification model to predict labels of unlabeled nodes based on our previous theoretical work [26]. We present an intuitive interpretation of the graph representation, label propagation and model formulation. We additionally analyzed the performance of the method in predicting the (sub)cellular localization of proteins. This method produced results that were both competitive and promising when compared to the state-of-the-art SVM classifier.

## Results

Our random walk classifier (RaWa) was coded in MATLAB. Given the training data and their classes, we computed the state matrix *Y *and weight matrix *W*. In our experiment, the similarity or weight between two nodes was given according to the radius basis function (RBF)

$$sim\left(v_{i},v_{j}\right)=e^{-\gamma {∥x_{i}-x_{j}∥}^{2}}=e^{-\frac{{∥x_{i}-x_{j}∥}^{2}}{2\sigma^{2}}}$$

To prove the effective classification performance of our method, we compared our classifier with RBF-SVM by implementing LibSVM [27], and the *γ *= 1/2*σ*^2 ^of our RaWa and RBF-SVM was optimized over the interval {2^-11^, 2^-9^, ..., 2^9^, 2^11^}. In this study, we adopted an *n*-fold cross-validation measurement to produce the highest predication accuracy, which was computed by dividing the number of correctly classified data points by the size of the entire unlabeled dataset.

### Predicting the (sub)cellular localization of proteins

Since our classifier involved two parameters, the laziness parameter *α *for constructing transition matrix and the random walk step *t*, we first tested the performance of our classifier on different combinations of *α *and *t*. Then, under the optimized parameter settings, we compared our approach with various measurements to the SVM classifier.

#### Influence of *α *and *t*

**Figure 1:**
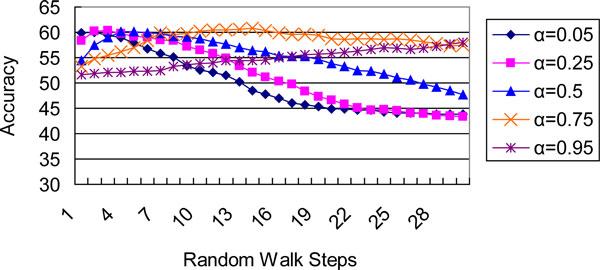
**Classification accuracies (in %) of yeast data given varying random walk steps and laziness parameters**.

**Figure 2:**
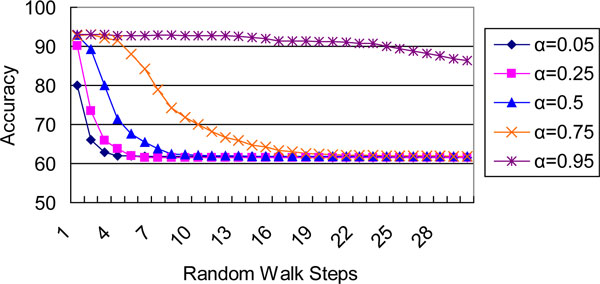
**Classification accuracies (in %) of gram-negative bacteria data given different random walk steps and laziness parameters**.

We investigated a maximum walk of 30 steps and five parameters: 0.05, 0.25, 0.5, 0.75 and 0.95. Figure 1 and Figure 2 depict the predictive accuracy curves of our random walk classifier on yeast and Gneg datasets, respectively. Each figure contains five lines that correspond to each *α *and depicts the trend of accuracy ratios with increasing *t*. The test results were obtained from 10-fold cross validation.

We found that a large number of steps were unnecessary for the RaWa classifier to achieve the best results. First, the complete graph offers each label a chance to reach the unlabeled node in at least one step. Second, both figures show that good accuracy was always obtained when the value of *t *was low. In contrast, the accuracy gradually declines after the peak value of *t*. This decline may probably due to the fact that with the increasing of *t*, *P^t ^*will become trivial and in turn mislead the classification. This situation is quite apparent in Figure 2. In addition, Szummer and Jaakola [28] found that small constant values of *t *(about *t *= 8) were effective on a dataset with several thousand examples.

Since the labeled training data is often deterministic, the transition matrix built over the labeled data is commonly treated as a unit matrix in semi-supervised random walk methods. However, the best result for the yeast data was achieved when *α *= 0.75. This value gave the labeled nodes more freedom to move to each other, whereas the best result for the Gneg data was achieved when *α *= 0.95. Consequently, it is necessary to import the laziness parameter when the training data is not fully reliable; *α *can usually be set above 0.5.

#### Comparisons with SVM

According to the above results, our method achieved a total prediction accuracy of 61% for yeast data, and >93% accuracy for Gneg data. Furthermore, to quantify the performance of our proposed algorithm, we employed SVMs and compared the two methods by computing the widely used measures of *Specificity *and *Sensitivity*. Table 1 compares the ability of the two methods to classify yeast data into 10 classes, while Table 2 shows the comparison for the Gneg data with 5 classes. We also compared the total accuracy of both classifiers; these data are presented in the final row of the table.

**Table 1:** Sensitivity and Specificity for yeast data using 10-fold cross-validation including the total predication accuracy

	RaWa		SVM	
	**Sensitivity**	**Precision**	**Sensitivity**	**Precision**

MIT	57.38	68.29	54.9	65.0
NUC	54.08	59.95	51.0	64.0
CYT	68.90	55.67	72.1	47.7
ME1	84.09	55.22	72.7	68.1
EXC	51.43	64.29	57.1	58.8
ME2	39.22	57.14	41.2	52.5
ME3	77.91	74.71	81.6	76.4
VAC	0	-	0	-
POX	55.00	84.62	0	-
ERL	1	83.33	0	0

Total Accuracy	61.3±0.11		60.2±0.28	

**Table 2:** Sensitivity and Specificity for gram-negative bacteria data using 10-fold cross-validation including the total predication accuracy.

	RaWa		SVM	
	**Sensitivity**	**Precision**	**Sensitivity**	**Precision**

Cytoplasm	89.3	94.0	93.6	85.6

Extracell	82.4	91.0	83.8	86.1

Inner membrane	98.2	93.7	95.9	96.5

Outer membrane	85.6	89.2	84.5	90.1

periplasm	79.3	91.1	84.5	85.2

Accuracy	93.3±0.24		92.1±0.46	

Each classifier was able to produce results with high sensitivity and specificity, but neither could identify the proteins that localized to the VAC site. The RaWa performs slightly better since it could predict the proteins that localized to POX and ERL, whereas the SVM could not. As illustrated in Table 2, both classifiers produced high sensitivities and specificities on the 5 locations, but according to the total accuracy listed in the last row, our classifier outperformed the SVM by 1%.

**Figure 3:**
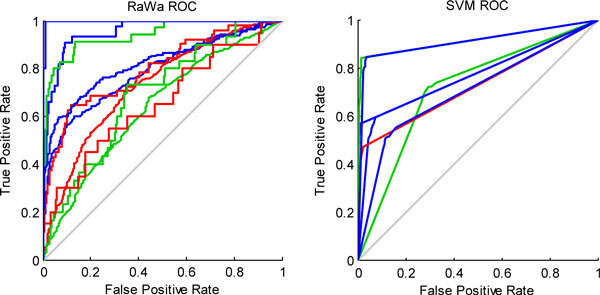
**ROC curves illustrating the comparison of RaWa and SVM methods on data from yeast**.

**Figure 4:**
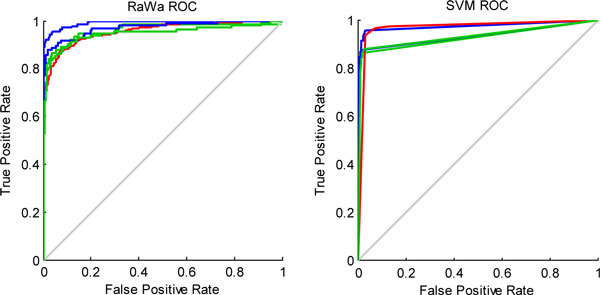
**ROC curves illustrating the comparison of RaWa and SVM methods on data from gram-negative bacteria**.

We further compared the two classifiers using receiver operating characteristic curves (ROCs). Figure 3 and Figure 4 depict the results for yeast and Geng, respectively, and each figure contains the ROC curve for the RaWa method on the left and the ROC curve for the SVM method on the right. These figures together offer an intuitive comparison and show that our RaWa classifier is effective and that the results are comparable to those derived from a SVM-based method.

## Discussion

Herein, we propose a novel classification model for label propagation through random walks on graphs. We first initialized an undirected complete graph over the labeled data whose data points act as the nodes and pairwise distances act as the weights. Then, labels and weights are employed to construct the state matrix and state transition matrix so that any node can start a random walk and propagate its label to any unlabeled data point after several steps. This model is also optimized by a kernel method and regularization so as to provide flexible control over the transition matrix.

One interesting possibility for future work is to develop algorithms for a clever selection of the labeled dataset and the kernel based on the data. In this study, we used the very simple Gaussian kernel with the identity covariance matrix, which likely does not exploit the similarity information conveyed in the data points.

## Conclusions

Protein cellular and subcellular localization has been an important facet of research because of its role in characterizing protein functions and protein-protein interactions. In this study, we developed a novel approach based on a random walk technique to predict protein localization. We demonstrated that this approach improves the accuracy of predicting protein (sub)cellular localization and is easy to train. When compared to the SVM classifier, our results are both competitive and promising.

## Methods

### Data preparation

To apply our method to predict and classify protein (sub)cellular localization, we utilized two datasets: the widely used yeast data from the UCI database and the gram-negative bacteria proteins from the Cell-PLoc package. The yeast data, including 1484 items with 8 attributes, were used to predict the cellular localization of proteins and have been categorized into 10 classes. The second dataset was first used by Shen and Chou in their predictors [29,30] particularly for the prediction of gram-negative bacteria proteins. This dataset contained 1114 gram-negative (Gneg) bacterial proteins classified into 5 subcellular locations according to experimental annotations. None of the proteins had more than 25% sequence identity to any other in the same subset (subcellular location). Detailed information is provided in Table 3.

**Table 3:** Information about gram-negative and yeast data

Proteins	Site	Number
Gram-negative bacteria proteins	Cytoplasm	140
	
	Extracellular	74
	
	Inner membrane	687
	
	Outer membrane	97
	
	Periplasm	116

Yeast	Cytosolic or cytoskeletal (CYT)	463
	
	Nuclear (NUC)	429
	
	Mitochondrial (MIT)	244
	
	Membrane protein, no N-terminal signal (ME1)	163
	
	Membrane protein, uncleaved signal (ME2)	51
	
	Membrane protein, cleaved signal (ME3)	44
	
	Extracellular (EXC)	37
	
	Vacuolar (VAC)	30
	
	Peroxisomal (POX)	20
	
	Endoplasmic reticulum lumen (ERL)	5

First, we represented a protein sample P with *L *amino acid residues by its evolutionary and sequence information. Here, for simplifying the formulation without losing generality, we use the numerical codes 1, 2... 20 to represent the 20 native amino acid types according to their single character symbols in alphabetical order. Then, the position-specific scoring matrix (PSSM) was introduced as a descriptor of evolutionary information. The PSSM produced a matrix M*_L×20 _*where M*_i→j _*represents the score of the amino acid residue in the *i*th position of the protein sequence being mutated to amino acid type *j *through evolution.

However, according to the PSSM descriptor, proteins with different lengths will correspond to matrices with different numbers of rows. To allow the PSSM descriptor to have a uniform representation, a given protein sample P could be represented by the mean value of each row: ${\bar{P}}_{PSSM}={\left[{\bar{M}}_{1},{\bar{M}}_{2},\cdots {\bar{M}}_{20}\right]}^{T}$

$${\bar{M}}_{j}=\frac{1}{L}\overset{L}{\underset{i=1}{\sum }}M_{i\to j}\left(j=1,2,\cdots \;,20\right)$$

However, as a result, all the sequence-order information would be lost. To avoid the complete loss of the sequence-order information, we also adopted the concept of the pseudo-amino acid composition (PseAA), as originally proposed in [31]. According to the representation of the PseAA, the protein P is formulated by

$$P_{PseAA}^{\lambda }={\left[p_{1},p_{2},\cdots \;,p_{20},p_{20+1},p_{20+2},\cdots \;,p_{20+\lambda }\right]}^{T}\left(\lambda <L\right)$$

where *p*_1_,*p*_2_,...,*p*_20 _are associated with the conventional amino acid composition, reflecting the occurrence frequencies of the 20 native amino acids in the protein P.

We thus represented the protein P by combining PSSM and PseAA in the following form $FT={\left[{\bar{P}}_{PSSM},P_{PseAA}^{\lambda }\right]}^{T}$.

In order to obtain the PseAA values, the lambda was set to 49, and the weight was 0.05. Since there are 3 proteins whose lengths were shorter than 49 amino acids, we obtained 1111 proteins with 89 features.

### Problem formulation

Usually, a training set (*X*, *C*) specifies the set of labeled data and the set of their classes, *n *is the number of tuples in *X*, and then the classes of a test set can be predicted. We first considered an initial graph of the form *G*(*V*, *E*, *W*), which was constructed over the training set, where *V *is the set of nodes and its member *v_i _*only responds to (*x_i_*, *c_i_*). This graph is assumed to be complete; therefore the edge set *E *is trivial. We thus provided the labeled nodes with a certain probability to travel to other nodes (explained below). *W *represents the edge weight matrix sized *n*×*n *and indicates the pairwise similarities, *w_ij _*= sim(*v_i_*,*v_j_*) = sim(*x_i_*,x_*j*_).

We also let *Y *be a set of *m *labels that can be applied to nodes of the graph. After the initial weighted graph was generated, a state transition matrix *P *= [*P_ij_*]*_n×n _*was defined to infer the probability *p_ij _*that one node *v_i _*transitions to the state of node *v_j_*. *P *is generally computed as *P *= *D*^-1^*W*, where the diagonal matrix *D *= diag(*W***1**_n_) and **1**_n _is a *n*-dimensional vector with all values set to 1. We next converted *y_i _*into a vector of labels (i.e., $Y={\left[y_{1},y_{2},...,y_{n}\right]}_{m\times n}$), where *y_i _*= [*y*_1*i*_,*y*_2*i*_,...,*y_mi_*]*^T^*. Therefore, the label or state of *v_i _*is c*_j _*if and only if *y_ji _*= 1. *Y *can be also referred to as the state matrix of *V *or *X*.

Given the state matrix and transition matrix, a simple random walk on *V *is described as the process that the state *y_i _*of any node *v_i _*transitions with the probability *p_ij _*to the state *y_j _*of node *v_j_*. Thus, the states of labeled data are not encoded as the absorbing states. Random walks on readily labeled nodes are meaningless since we utilized the information already encoded in the partially labeled graph to help us predict labels, but the initial graph *G *is just a labeled graph. Therefore, given each data point lacking a label from the test set, we added it to graph *G *as an unlabeled node. The traditional classification problem has thus been converted to a node classification problem on a partially labeled graph by this method.

### Random walk classification model

We next aimed to deduce a simple classifier based on the nodes that are labeled so it can be applied to predict the labels of the unlabeled nodes. Our solution was a state vector *y *that provides the label for an unlabeled data point *x*.

**Figure 5:**
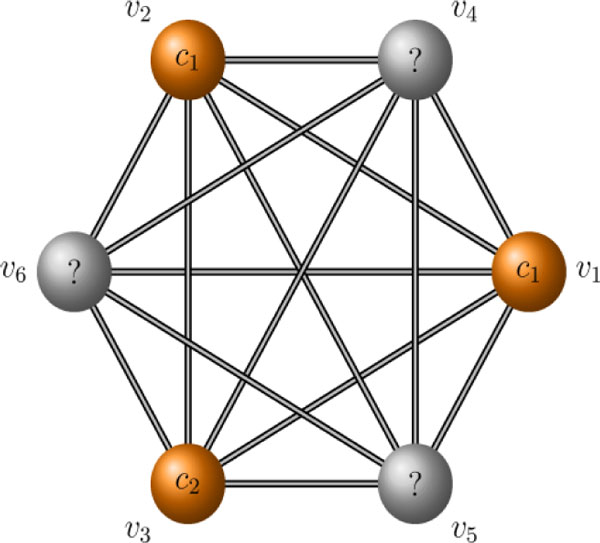
**A simple partially labeled graph**.

We first provide an example to clarify the process of label propagation through random walks. Consider an initial graph *G *constructed over the training data (*X*, *Y*) = {(*x*_1_, *c*_1_), (*x*_2_, *c*_1_), (*x*_3_, *c*_2_)}. Each data point lacking a label is added into graph *G *as an unlabeled node. Figure 5 displays such a graph *G' *after three unlabeled data points were added. The graph *G' *is often assumed to be label-connected to become completely labeled [32]; that is, it is possible to reach a labeled node from any unlabeled node in a finite number of steps. For example, if in a random walk, the sixth node *v*_6 _ends at the second node *v*_2_, then this node will be labeled as *c*_1_.

Node classification relies on a random walk originating at the unlabeled node *v_j _*and ends at one labeled node *v_i _*after several steps, and in this way, *v_j _*obtains its label from *v_i_*. If during the walk an unlabeled node reaches a labeled node for the first time, it will not remain at that node because the labeled nodes are not absorbing states; rather, the unlabeled node will move to another node with a certain probability. Since graphs *G *and *G' *are undirected and symmetric, a random walk that starts at *v_j _*and ends at *v_i _*can be also revertible.

Next, we assume *p*(*v_i_*, *v*) to be the state-transition probability with which a walk proceeds from node *v_i _*in *V *to the new node *v *represented by unlabeled data point *x*. The state *y *of new node *v *is represented as

$$y=\underset{v_{i}\in V}{\sum }p\left(v_{i},v\right)y_{i}=Yp_{v}$$

where

$$p_{v}\overset{def}{=}p\left(V,v\right)=\left[\begin{array}{c} p\left(v_{1},v\right)\\ p\left(v_{2},v\right)\\ \vdots \\ p\left(v_{n},v\right) \end{array}\right]$$

For the node *v_i _*in *V*, we have

$$p\left(V,v_{i}\right)=D^{-1}w_{i}=D^{-1}\left[\begin{array}{c} w_{1i}\\ w_{2i}\\ \vdots \\ w_{ni} \end{array}\right]$$

Similarly, for the new node *v *not in *V*, *p*(*V*, *v*) is computed as:

$$p\left(V,v\right)=D^{-1}w\left(V,v\right)=D^{-1}\left[\begin{array}{c} w\left(v_{1},v\right)\\ w\left(v_{2},v\right)\\ \vdots \\ w\left(v_{n},v\right) \end{array}\right]$$

Therefore, the state *y *of *v *can be obtained by the following equation:

$$\begin{array}{llll} y & =Yp\left(V,v\right)\; & & \;\\ & =YD^{-1}w\left(V,v\right)\; & & \;\\ & =YD^{-1}WW^{+}w\left(V,v\right)\; & & \;\\ & =YPW^{+}w\left(V,v\right)\; & & \;\\ & \; & \end{array}$$

where *W*^+ ^denotes the pseudo-reverse matrix of *W*. This is preferred over the inverse of *W *because *W *may sometimes be singular. *w*(*V*, *v*) is a column vector that indicates the similarity between the new node *v *and nodes in *V*.

### Model training

In order to train an effective classifier, the labeled data should be fully utilized; however the influence of noise within the training data should be avoided, especially because biological measurements always contain a certain amount of noise.

Therefore, we trained our classification model with a prediction adjustment using complementary training data. We first partitioned the training data *X *in a balanced fashion, which resulted in two subsets with a similar size, each having a certain amount of data belonging to each class in *C*. The two subsets *S *and *T *thus have properties such that *S*∪*T *= *X *and *S*∩*T *= *Φ*. Next, we allow the two complementary sets to predict each other with the above equation, and we can get:

$$F_{S}\left(T\right)=Y_{S}P_{S}W_{S}^{+}w\left(S,T\right)$$

$$F_{T}\left(S\right)=Y_{T}P_{T}W_{T}^{+}w\left(T,S\right)$$

To evaluate the performance of this prediction, we computed the test loss on *S *and *T *according to the following equations:

$$\varepsilon_{F_{S}}=\varepsilon \left(F_{S},X,T\right)={∥Y_{S}P_{S}W_{S}^{+}w\left(S,T\right)-Y_{T}∥}_{F}^{2}$$

$$\varepsilon_{F_{T}}=\varepsilon \left(F_{T},X,S\right)={∥Y_{T}P_{T}W_{T}^{+}w\left(T,S\right)-Y_{S}∥}_{F}^{2}$$

where classifier's performance increases with decreasing test loss. Moreover, we defined the total loss as

$$\varepsilon_{F_{X}}=\frac{\varepsilon_{F_{S}}\varepsilon_{F_{T}}}{\varepsilon_{F_{S}}+\varepsilon_{F_{T}}}$$

Though the total loss could be minimized through repeated random partitions of the training data, it is time consuming. We note that the test loss also indicates the importance of its corresponding subset, so we can impose a weight on each subset to highlight this difference. We then defined the state matrix to be:

$$Ỹ=\vec{\alpha }Y=\left[\alpha_{S}Y_{S},\alpha_{T}Y_{T}\right]\;s.t.\;\alpha_{S}+\alpha_{T}=1$$

The weight vector was computed as follows:

$$\vec{\alpha }=\left[\begin{array}{c} \alpha_{S}\\ \alpha_{T} \end{array}\right]=\frac{1}{\varepsilon_{F_{S}}+\varepsilon_{F_{T}}}\left[\begin{array}{c} \varepsilon_{F_{T}}\\ \varepsilon_{F_{S}} \end{array}\right]$$

For the transition matrix, we usually consider a multi-step random walk; for *t *steps, we just replace *P *with *P^t^*. During a random walk of *t *steps, the state of the new node *v *or new data point *x *is:

$$y=\vec{\alpha }YP^{t}W^{+}w\left(V,v\right)$$

Previous studies have treated the labeled nodes as absorbing states, such that *P *= *I*, but here we considered lazy random walks, i.e., *P^t ^*= (*α*I + (1-*α*)*P*)*P^t^-1*, where *α*∈(0,1) is a laziness parameter indicating that the nodes will stay at their current positions with probability *α*

### Further improvement with the kernel method and regularization

Usually, *k*(*u*, *v*) denotes the kernel function so that *k*(*X*, *x*)=[*k*(*x*_1_, *x*), *k*(*x*_2_, *x*),..., *k*(*x_n_*, *x*)]*^T^*. We defined the kernel matrix *K *in the space (*X*, *X*) as *K *= *k*(*X*, *X*) = [*k*(*x_i_*, *x_j_*)]*_n×n_*, and *F *was defined as a classifier. The kernel function *k*(*X*, *x*) and kernel matrix *K *were employed to substitute for the similarity metric *w*(*V*, *v*) and weighted matrix *W*, respectively.

$$w_{ij}\overset{def}{=}w\left(v_{i},v_{j}\right)=k\left(x_{i},x_{j}\right)$$

With the kernel method embedded, we formulated our random walk classifier as:

$$F\left(x\right)=ỸP^{t}K^{+}k\left(X,x\right)$$

Again, assuming $\hat{F}=ỸP^{t}$, the final classification model is represented as:

$$F\left(x\right)=\hat{F}K^{+}k\left(X,x\right)$$

The idea underlying the random walk methods is that the probability of labeling a node *v *with a label (or state) *y *is the total probability that a random walk starting at *v *will end at a node labeled *y*. *F*(*x*) therefore is more likely to return a probability distribution such as $F\left(x_{i}\right)=F\left(v_{i}\right)={\left[f_{1i},f_{2i},...,f_{ci}\right]}^{T}$, where each distribution *f_ji _*refers to the total probability that the a random walk starting at node *v_i_*stops at any node labeled *c_j _*after *t *steps. The largest *f_ji _*allows *v_i _*to be assigned label *c_j_*.

$$f_{ji}=\underset{v_{i}\in V|y_{ji}=1}{\sum }p_{ij}$$

*K *sometimes is a singular matrix because of insufficient data or the existence of noise, or there could be more than one optimized solution for *W*. In either case, computing *w *is not recommended. We thus use regularization to improve upon ill-posed problems. To enhance the robustness of our classifier, we introduced a regularization parameter *λ *into the kernel matrix, thereby formulating the regularized random walk basic classifier. In our experiments, we fixed *λ *to 0.0001 to avoid interference from the original data.

$$F\left(x\right)=\hat{F}{\left(K+\lambda I\right)}^{+}k\left(X,x\right)$$

If the dimension of *X *is *d*, then the time cost for computing the kernel matrix and pseudo-reverse matrix to build the model for our classifier is *O*(*dn*^2^) and *O*(*n*^3^), respectively. $\hat{F}K^{+}$ requires a complexity of *O*(*mn*^2^), where *m *≤ *n*, so the overall cost is estimated as *O*(*dn*^2^) + *O*(*n*^3^) + *O*(*mn*^2^) = *O*(max{*d*, *n*}*n*^2^).

## List of Abbreviations

HMM: Hidden Markov Models; kNN: k Nearest Neighbor; SVM: Support Vector Machine; RBF: Radial Basis Function; PSI: Position-Specific Iterated; BLAST: Basic Local Alignment Search Tool; PseAA: Pseudo Amino acid; RaWa: Random Walk Classifier; Gneg: gram-negative bacteria; ROC: receiver operating characteristic curve.

## Authors' contributions

XX conceptualized the theoretical framework for this study. LL implemented the idea and conducted the experiments. PH, LC managed and coordinated the project. All authors participated in writing and revising the final manuscript.

## Competing interests

The authors declare that they have no competing interests.

## Declarations

This article has been published as part of *BMC Bioinformatics *Volume 14 Supplement 8, 2013: Proceedings of the 2012 International Conference on Intelligent Computing (ICIC 2012). The full contents of the supplement are available online at http://www.biomedcentral.com/bmcbioinformatics/supplements/14/S8.
